# Relationship of Composite Dietary Antioxidant Index vs. Alcohol Consumption with Mild Cognitive Impairment in the Elderly

**DOI:** 10.3390/nu17132111

**Published:** 2025-06-25

**Authors:** Mengjie He, Yan Zou, Danting Su, Dong Zhao, Mengyi Zhou, Peiwei Xu, Ronghua Zhang

**Affiliations:** 1 Department of Nutrition and Food Safety, Zhejiang Provincial Center for Disease Control and Prevention, Hangzhou 310051, China; mjhe@cdc.zj.cn (M.H.); yzou@cdc.zj.cn (Y.Z.); dtsu@cdc.zj.cn (D.S.); dzhao@cdc.zj.cn (D.Z.); pwxu@cdc.zj.cn (P.X.); 2 NHC Specialty Laboratory of Food Safety Risk Assessment and Standard Development, Hangzhou 310051, China; 3 School of Public Health, Hangzhou Medical College, Hangzhou 310053, China; 15058972559@163.com

**Keywords:** composite dietary antioxidant index, alcohol consumption, mild cognitive impairment, interactive effect

## Abstract

**Background/Objectives**: Precise prevention strategies for mild cognitive impairment (MCI) are an urgent public health priority. This study aimed to investigate the association of the Composite Dietary Antioxidant Index and alcohol consumption, as well as their interaction, with the risk of MCI. **Methods**: A multicenter cross-sectional study was conducted in 2020, involving 1084 individuals aged ≥55 years, in Zhejiang Province, China. Data were collected on demographics, cognitive function, alcohol consumption, depression scale, dietary intake and physical examinations. The Composite Dietary Antioxidant Index (CDAI) was calculated based on the converted Food Frequency Questionnaire (FFQ) Food Composition Tables and the data of the FFQ. CDAI values were divided into four groups by interquartile ranges: Quartile-1 (Q1), Quartile-2 (Q2), Quartile-3 (Q3) and Quartile-4 (Q4). Multivariate logistic regression models were used to evaluate the association of CDAI and alcohol consumption with MCI risk and their interaction. **Results**: The prevalence of MCI was 24.6%. After adjusting for gender, age, educational level, job, marriage, Body Mass Index (BMI), central obesity, frequency of social activities, depression, sleep disturbances, smoking, diabetes, and energy intake, the Q2 (OR = 0.63, 95% CI: 0.42~0.95), Q3 (OR = 0.52, 95% CI: 0.34~0.81) and Q4 (OR = 0.25, 95% CI: 0.14~0.48) of CDAI were significantly associated a reduced risk of MCI. In contrast, alcohol consumption 1~2 times per week (OR = 2.38, 95% CI: 1.02~5.59) and at least 3 times a month (OR = 2.04, 95% CI: 1.19~3.50) was significantly associated with an increased risk of MCI. Interaction analysis indicated a negative additive interaction between alcohol consumption and CDAI on MCI risk, with the detrimental effects of alcohol predominating. **Conclusions**: A higher CDAI is associated with a reduced risk of MCI, while alcohol consumption is associated with an increased risk. There may be a negative additive interaction between CDAI and alcohol intake in relation to MCI. Targeted strategies that reduce alcohol consumption and improve dietary antioxidant intake are essential for MCI prevention.

## 1. Introduction

Global population aging is accelerating at an unprecedented pace, including in China [1,2]. Alzheimer’s disease (AD), the leading cause of dementia associated with aging, imposes a substantial burden on families, healthcare systems, and societies worldwide [3,4,5]. Globally, it is estimated that 55 million individuals are living with AD, including approximately 10 million in China [4,5]. Despite extensive research, no clinical trial for AD treatment has achieved success to date [6], underscoring the urgent need for primary prevention strategies. Mild cognitive impairment (MCI) represents an intermediate state between normal age-related cognitive decline and dementia, with 10–20% of individuals with MCI progressing to dementia within 12 months [7]. Identifying modifiable risk factors, high-risk groups, and protective factors for MCI is essential for developing targeted AD prevention strategies.

Previous studies have suggested that alcohol consumption might be a strongly modifiable risk factor for MCI [8,9,10,11]. Alcohol induces oxidative stress in the brain by increasing free radical production and weakening antioxidant defenses, thereby impairing neuronal function and precipitating cognitive impairment [12]. While excessive alcohol consumption has well-documented negative effects, some studies have reported that low-to-moderate intake may offer protective benefits [13,14]. Therefore, the relationship between alcohol consumption and MCI remains inconclusive and warrants further investigation. Nutrition plays a crucial role in brain senescence. Certain nutrients with antioxidant properties may protect against MCI [15]. The Composite Dietary Antioxidant Index (CDAI) is validated tool for assessing the overall antioxidant capacity of daily diets [16]. It integrates scores from six key antioxidant nutrients: vitamins A, E, C, zinc, selenium, and magnesium [17]. Previous studies have reported the association between CDAI and chronic diseases, including cardiovascular and cerebrovascular diseases and chronic obstructive pulmonary disease [18,19]. Data from NHANES (2011–2014) also demonstrated that higher CDAI levels were significantly associated with improved cognitive function. However, no studies to date have investigated the association between CDAI and the risk of developing MCI. In addition, the relative impact of alcohol-induced oxidative stress versus dietary antioxidant capacity on MCI risk remains undefined. This study aimed to explore preventive strategies for the pre-dementia stage of AD. Specifically, this multicenter cross-sectional study investigated the associations of alcohol and CDAI with MCI, along with their potential interaction, to provide a scientific basis for MCI prevention.

## 2. Materials and Methods

### 2.1. Study Design

The data used in this study were derived from follow-up evaluations of the Community-based Cohort Study on Nervous System Diseases-AD cohort [20]. This cohort primarily investigated potential risk factors associated with Alzheimer’s disease (AD) in adults aged 55 years and older. Detailed information was described previously [21]. In brief, a field survey was conducted in Zhejiang Province in September 2020. The study protocol received ethical approval by the Medical Ethics Committee of the National Institute for Nutrition and Health, Chinese Center for Disease Control and Prevention (Approval No. 2017020, 6 November 2017). In accordance with ethical guidelines, written informed consent was obtained from all participants prior to their enrolment in the study.

### 2.2. Study Population and Sampling Method

**Figure 1:**
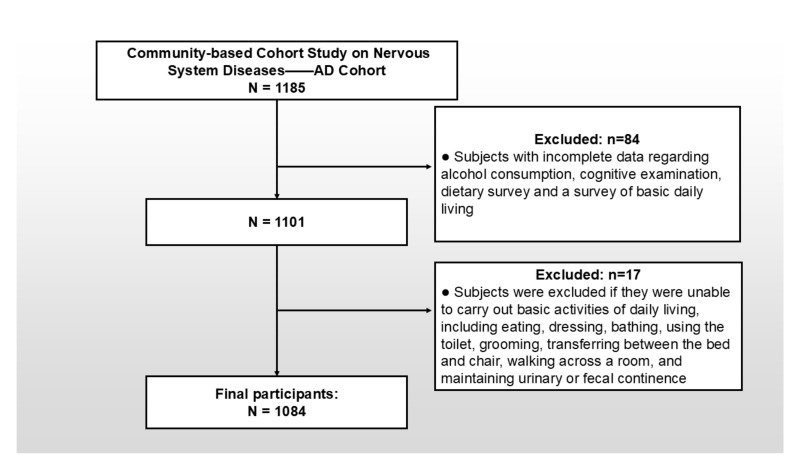
Flow chart of the analysis sample in this study.

The study cohort was established using a multistage stratified random sampling approach during the baseline survey implemented in Zhejiang Province [21]. The inclusion criteria for participants in this study were as follows: (1) individuals aged 55 years or older; (2) permanent residency in the selected community; and (3) absence of comorbidities that could confound the assessment results, such as congenital or acquired intellectual disability and uncorrectable visual or auditory impairments. After applying these criteria and conducting rigorous quality control procedures, a total of 1084 participants were included in the final analytical sample, as depicted in Figure 1.

### 2.3. Data Collection

Data collection was conducted through two main approaches: (1) a questionnaire was used to assess sociodemographic characteristics (age, educational level, occupation, and marital status), cognitive function, alcohol consumption, other health-related information, and dietary intake. Dietary intake was measured using a validated 81-item Food Frequency Questionnaire (FFQ) encompassing various food categories; (2) standardized physical examinations were carried out by qualified health professionals from local community health centers, including measurements of blood pressure, height, weight, and waist circumference. Rigorous quality control measures were implemented both before and during the investigation to ensure data accuracy and reliability [21].

### 2.4. Assessment of Cognitive Function

Given the advantages of the Montreal Cognitive Assessment (MOCA) over the Mini-Mental State Examination (MMSE), this study employed the MOCA scale exclusively to assess participants’ cognitive function [22]. The Beijing version of the MoCA scale, which has demonstrated reliability and validity in the Chinese population, was adopted [23,24,25]. Standardized face-to-face interviews were conducted by trained investigators, who followed established testing procedures and administration guidelines. MCI was diagnosed based on the Chinese norms for the MoCA [23].

### 2.5. Assessment of Composite Dietary Antioxidant Index and Energy

Dietary nutrient intakes were assessed according to the FFQ, including 64 categories of foods and 7 types of nutrient supplements, and converted FFQ Food Composition Tables (FCTs). Since the FFQ in this study was the same as that in the China Nutrition and Health Project, the data from the China Health and Nutrition Survey (CHNS) in Zhejiang province (2018) [26] were utilized to develop the converted FFQ FCTs. Previous studies have demonstrated that the Weighted Food Composition Table method (WFCT) is optimal for nutrient assessment in FFQ-based studies [27]. Consequently, the WFCT was applied to establish the converted FFQ FCTs. This was achieved using representative 3-day 24 h dietary recall (24HRs) data and the Chinese Food Composition Table (2019) [28]. Based on the converted FFQ FCTs and the FFQ employed in this study, daily per capita energy and nutrient intake were calculated. The CDAI was computed using dietary intakes of six antioxidants (vitamin A, vitamin E, vitamin C, zinc, selenium and magnesium) [17]. The calculation formula is as follows [16].

$$\mathrm{CDAI}=\sum_{i=1}^{n=6}\frac{Individual\;Intake-Mean}{SD}$$

The CDAI was divided into four groups by interquartile range (IQR), including Quartile-1 (Q1), Quartile-2 (Q2), Quartile-3 (Q3) and Quartile-4 (Q4).

### 2.6. Assessment of Alcohol Consumption and Other Covariates

Alcohol consumption was divided into four groups, which were no consumption, less than once a month, 1~10 times a month, at least 3 times per week, in the past one year, respectively. Participants with a previous diagnosis of hypertension, or those whose average systolic blood pressure measured three times during a physical examination was >140 mmHg or whose average diastolic blood pressure measured three times was >90 mmHg, were defined as the hypertension population. Depressive symptoms were assessed using the validated Geriatric Depression Scale (GDS) [29]. Central obesity was defined in line with the criteria for adult weight in China [30]. The subjects were divided into the following three groups according to Body Mass Index (BMI): <24.0 kg/m^2^ and ≥24 kg/m^2^ [31]. Sleep disturbance was evaluated using the Chinese-validated version of the Pittsburgh Sleep Quality Index (PSQI), for which a cut-off score of 5 was recommended as the screening criterion [32,33]. Smoking was defined as “Yes” if the participant had smoked and “No” if the participant had never smoked.

### 2.7. Statistical Analyses

Mean and standard deviations (mean ± SD) were employed to describe continuous variables with a normal distribution, while median and quartiles [(median (quartiles)] were utilized for variables presenting a skewed distribution. Frequencies and percentages (%) were reported for categorical variables. Categorical variables were compared using the chi-square test. Multiple logistic regression models were established to examine the effect of CDAI and alcohol consumption on MCI. The odds ratio (OR) and the corresponding 95% confidence interval (CI) of the potential influencing factors, including gender, age, educational level, job, marriage, BMI, central obesity, frequency of social activities, depression, sleep disturbances, smoking, diabetes, energy intake, and hypertension, were calculated using multivariate-adjusted models. To test for the interaction effect, multivariate-adjusted multiple logistic regression models were constructed, which were used to perform a subgroup analysis of CDAI in relation to the impact of alcohol consumption on MCI. To investigate the interactive effect of CDAI and alcohol consumption on MCI, joint alcohol consumption (No/Yes) and CDAI (Q1/Q2/Q3/Q4) variables were created [34]. Specifically, participants were classified into 8 groups according to both their alcohol consumption and CDAI. Multivariate-adjusted models were generated with the joint variables using multiple logistic regression models. The validity of the food and nutrient intake assessed by the Food Frequency Questionnaire was analyzed using Spearman’s correlation analysis. All statistical analyses were performed by using the program SAS version 9.1. *p* less than 0.05 was considered statistically significant.

## 3. Results

### 3.1. Basic Information

A total of 1084 participants were included in this study. The key characteristics are presented in Table 1. The overall prevalence of MCI was 24.6%. The study population was well distributed across regions, age groups, and gender. The Cochran–Mantel–Haenszel trend chi-square test revealed a significant trend of decreasing MCI prevalence with increasing CDAI (*p* = 0.0024).

### 3.2. Analysis of the Association Between Composite Dietary Antioxidant Index and MCI

**Figure 2:**
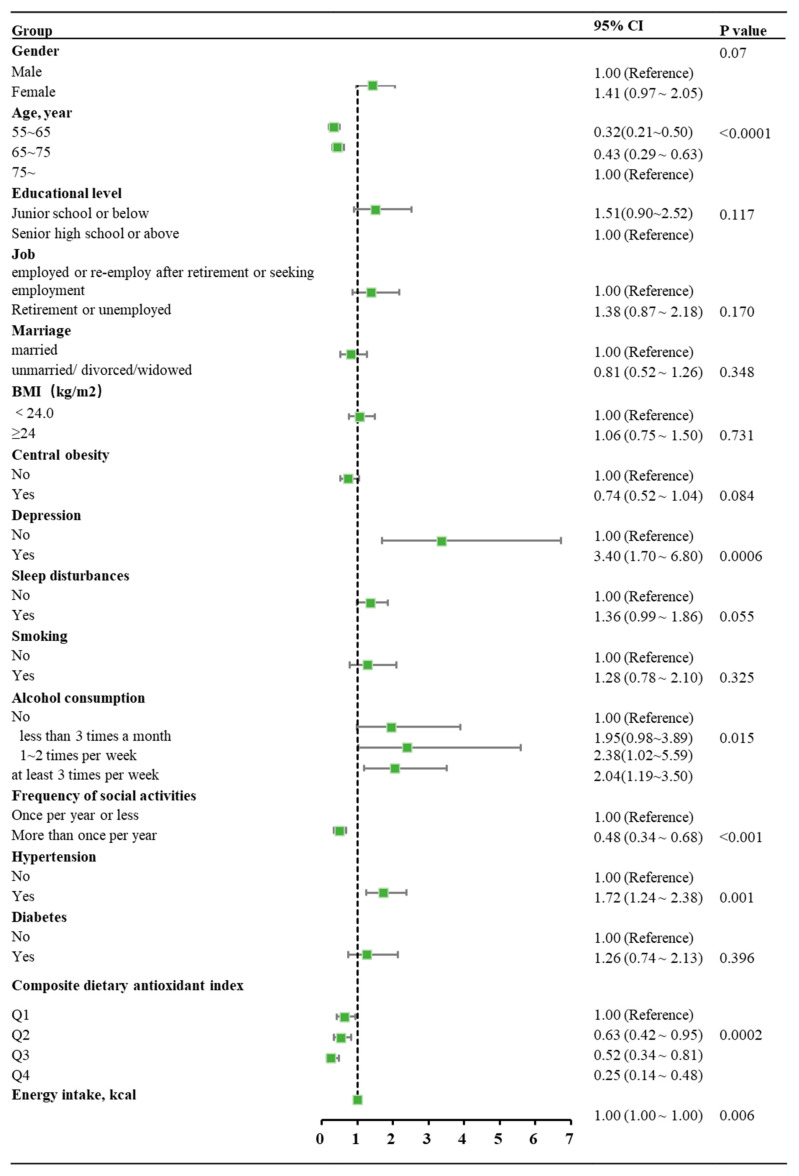
Forest plot of the risk of MCI by the multivariate logistic regression model. The green marks represent OR values, and the line segments represent 95% CI.

A total of 946 participants were included in the calculation of the converted FFQ FCTs. Daily nutrient intakes assessed by FFQ using the WFCT method demonstrated high correlations with those assessed by 24HRs in CHNS-2018 in Zhejiang Province, and detailed results are presented in Supplementary Table S1. Using the converted FFQ FCTs (see Supplementary Tables S2 and S3), dietary nutrients, including vitamin A, vitamin E, vitamin C, zinc, selenium, and magnesium, and energy intake were calculated. CDAI was calculated according to the intake of six dietary nutrients (vitamin A, vitamin E, vitamin C, zinc, selenium, and magnesium). CDAI and alcohol consumption, as well as gender, age, educational level, job, marriage, BMI, central obesity, frequency of social activities, depression, sleep disturbances, smoking, diabetes, energy intake and hypertension were included in the univariate chi-square analysis and the multivariate analysis, as shown in Table 1 and Figure 2, respectively. The multivariate analysis showed that Q2 (OR = 0.63, 95% CI: 0.42~0.95), Q3 (OR = 0.52, 95% CI: 0.34~0.81) and Q4 (OR = 0.25, 95% CI: 0.14~0.48) of CDAI were all linked to a significantly reduced risk of MCI. Moreover, the trend test indicated that there is a significant upward trend in the risk of developing MCI as the CDAI increases (*p* < 0.0001).

### 3.3. Analysis of the Association Between Alcohol Consumption and MCI

The multivariate analysis showed that alcohol consumption 1~2 times per week (OR = 2.38, 95% CI: 1.02~5.59) and alcohol consumption at least 3 times a month (OR = 2.04, 95% CI: 1.19~3.50) were linked to a significantly increased risk of MCI. However, alcohol consumption less than 3 times a month exerted no significant impact on the risk of MCI. Moreover, no significant upward trend was observed for the risk of developing MCI with an increasing frequency of alcohol consumption.

### 3.4. Interactive Effect of CDAI and Alcohol Consumption on MCI

**Figure 3:**
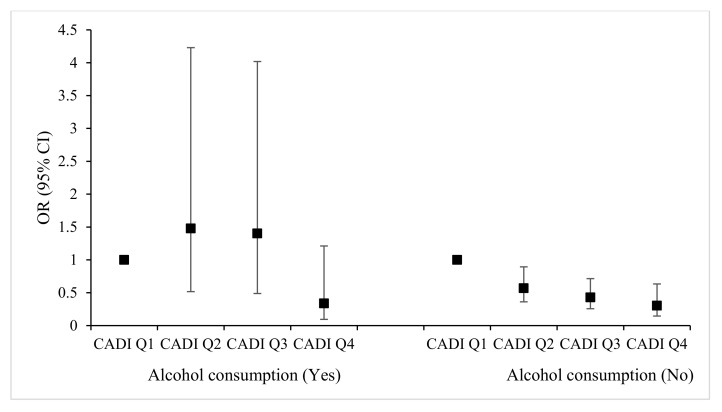
Joint relation of CDAI in quartiles vs. alcohol consumption with the odds ratio of MCI. The black squares represent OR values, and the black lines represent 95% confidence intervals.

Based on Figure 3, the OR for the effects of different alcohol consumption frequencies on MCI was similar. However, some groups had relatively small sample sizes. When assessing the interaction between alcohol consumption and CDAI on the MCI, alcohol consumption was dichotomized into two groups: “NO”, comprising individuals who abstained from alcohol in the past year, and “YES”, including those who had consumed alcohol within the past year. Subgroup analyses examined the association between CDAI in quartiles and MCI stratified by alcohol consumption, as shown in Figure 3. Multivariate analysis found that among non-drinkers, Q2 (OR = 0.57, 95% CI: 0.36~0.89), Q3 (OR = 0.43, 95% CI: 0.26~0.72) and Q4 (OR = 0.30, 95% CI: 0.15~0.63) of CDAI were all linked to a significantly reduced risk of MCI. Additionally, a significant upward trend in the MCI-development risk with increasing CDAI was observed. However, among participants who consumed alcohol, CDAI was not significantly associated with MCI. Subgroup analysis further demonstrated a significant interaction between CDAI and alcohol consumption on MCI risk (*p* < 0.05). The combined effect of CDAI and alcohol consumption is shown in Table 2. All no alcohol-consuming groups and the alcohol-consuming population in the highest CDAI (Q4) group had a statistically significant reduced risk of MCI compared with the no alcohol-consuming CDAI (Q1) reference group. Comparing alcohol-consuming and non-consuming groups within the CDAI Q4 category, the OR decreased from 0.346 to 0.316, suggesting a negative additive interaction between alcohol consumption and CDAI on MCI. Considering differences in CDAI distribution across alcohol consumption groups, as presented in Supplementary Table S4, the IQRs of CDAI were adjusted accordingly. The results of the multivariate analysis following the adjustment of threshold values, as depicted in Supplementary Table S5, were consistent with the overall pre-adjustment situation.

## 4. Discussion

In the absence of curative treatments for AD, implementing primary prevention strategies to identify MCI before the onset of AD is of utmost importance. This study aimed to investigate the association of CDAI and alcohol consumption with MCI, along with their interaction effects, thereby achieving precise prevention of MCI. In this study, the prevalence of MCI was 24.6%. Multivariate analysis revealed that participants in the second (Q2, OR = 0.63, 95% CI: 0.42–0.95), third (Q3, OR = 0.52, 95% CI: 0.34–0.81) and highest (Q4, OR = 0.25, 95% CI: 0.14–0.48) CDAI quartiles had significantly reduced risks of MCI compared to the lowest quartile. Moreover, a significant decreasing trend in MCI risk was observed with increasing CDAI levels. Conversely, an alcohol consumption frequency of 1–2 times per week (OR = 2.38, 95% CI: 1.02–5.59) and at least 3 times per month (OR = 2.04, 95% CI: 1.19–3.50) was significantly associated with increased risk of MCI. Subgroup analyses showed that among non-drinkers, higher CDAI was significantly associated with lower MCI risk, exhibiting a clear protective trend. However, among participants who consumed alcohol, CDAI was not significantly related to MCI risk. Interaction analyses demonstrated a significant effect between CDAI and alcohol consumption on MCI, with the adverse effect of alcohol consumption predominating.

The China Health and Retirement Longitudinal Study reported that a total of 3622 elderly individuals were assessed using the MMSE scale, revealing a cognitive impairment prevalence of 41.6% nationwide [35]. The lower prevalence of MCI in Zhejiang Province compared to the national average may be attributable to the region’s higher economic development level.

As shown in Supplementary Table S1, the Spearman correlation coefficients were consistent with those in previous validations of the FFQ [27], supporting the use of the Converted FFQ Food Composition Tables for estimating nutrient intakes and calculating CDAI. Both the CDAI and alcohol consumption data in this study were collected retrospectively, covering the year prior to MCI diagnosis, thus maintaining the appropriate temporal sequence for causal inference. Emerging evidence from multiple analyses of the NHANES study has demonstrated a potential protective effect of higher CDAI on cognitive function [36,37,38]. The reported odds ratios for CDAI and cognitive function impairment were 0.81 in Q2, 0.69 in Q3, and 0.59 in Q4. Similarly, a prospective cohort study in Singapore reported an odds ratio (95% CI; p-trend) of 0.84 (0.73, 0.96; p-trend = 0.003) when comparing the highest with the lowest CDAI quartile. In the present study, the observed ORs of the CDAI and MCI were lower than those in the aforementioned studies. To date, limited investigations have explored the association between CDAI and MCI specifically. Given the role of alcohol consumption in inducing oxidative stress [39], this study included alcohol consumption as a variable to investigate its interaction with CDAI on MCI. Initial research on alcohol consumption and cognition laid the groundwork for establishing drunk driving regulations [40]. Subsequent studies suggested a potential “J-shaped” or “inverse U-shaped” relationship between alcohol consumption and cognitive decline in the progression of normal cognition to MCI or MCI to AD [8,10,11]. Mitigating MCI risk in populations with excessive alcohol use remains important. Interaction analysis revealed a negative interaction between CDAI and alcohol consumption on MCI risk. Previous research has highlighted the neuroprotective effects of dietary antioxidants—such as selenium and vitamin C—on cognitive function [41,42]. However, alcohol-induced oxidative stress may diminish this protective effect. Interestingly, when CDAI was in the fourth quartile, alcohol consumption did not appear to weaken its protective association with MCI, though no prior studies are available to confirm this finding.

Covariate analysis indicated that aging, depression symptoms, hypertension, and limited social activities were significantly associated with an elevated MCI risk. These findings are consistent with well-established risk factors for MCI or cognitive decline reported in previous studies, supporting the validity and reliability of the present data [11,43,44,45,46,47,48]. In univariate correlation analysis, education level and sleep disturbances were significantly associated with MCI. However, this association did not persist in the multivariate analysis. Given the close relationship between sleep disturbances and depression [49], multicollinearity between these variables may have influenced the results. Although previous studies [50,51,52] have found that educational level, BMI, marriage status, and smoking status influence MCI, no significant associations with these variables were observed in this study.

Our study encompasses several notable strengths. Firstly, to our knowledge, it is the first to investigate the relationship between CDAI and MCI. Secondly, it is also the first to examine the interaction between CDAI and alcohol consumption in relation to MCI risk. Third, the study developed and applied a converted Food Frequency Questionnaire (FFQ)-based Food Composition Table, allowing for more accurate assessment of dietary nutrient intake in the target population.

Notwithstanding its contributions, our study had several limitations, and the findings should be interpreted and generalized with caution. The cross-sectional design precludes any inference of causality, limiting the ability to determine temporal relationships between exposures and MCI. Second, dietary intake was self-reported, which may introduce recall bias and affect the precision of nutrient intake estimation. Third, the assessment of alcohol consumption lacked granularity. Important variables such as alcohol volume, specific drinking patterns, and history of abstinence were not collected, preventing a more nuanced quantitative analysis of alcohol exposure. Lastly, the study was conducted in a limited geographic area with a relatively small sample size, which may restrict the generalizability of the results. Therefore, future large-scale, longitudinal studies are warranted to validate and extend these findings.

## 5. Conclusions

In summary, this study found a prevalence of MCI of 24.6% among individuals aged ≥55 years in Zhejiang Province. A higher CDAI was significantly associated with a reduced risk of MCI, whereas alcohol consumption was associated with an increased risk. Moreover, a negative additive interaction between CDAI and alcohol consumption on MCI was observed, suggesting that alcohol intake may attenuate the protective effects of dietary antioxidants on cognitive health. These findings underscore the importance of limiting alcohol consumption as part of cognitive health maintenance strategies. Future prospective studies are needed to establish causal relationships and to develop targeted, evidence-based interventions for MCI prevention.

## Figures and Tables

**Table 1:** Main characteristics of study participants.

Variables		Total, *n*	MCI, *n* (%)	Normal, *n* (%)	χ^2^	*p*
Total		1084	267 (24.6)	817 (75.4)		
Composite dietary antioxidant index					**8.96**	**0.030**
	Q1	311 (28.7)	91 (34.1)	220 (26.9)		
	Q2	279 (25.7)	74 (27.7)	205 (25.1)		
	Q3	258 (23.8)	57 (21.4)	201 (24.6)		
	Q4	236 (21.8)	45 (16.9)	191 (23.4)		
Alcohol consumption					**12.40**	**0.0** **06**
	No	874 (80.6)	197 (73.8)	677 (82.9)		
	Less than 3 times a month	59 (5.4)	17 (6.4)	42 (5.1)		
	1~2 times per week	34 (3.1)	14 (5.2)	20 (2.5)		
	At least 3 times per week	117 (10.8)	39 (14.6)	78 (9.6)		
Region					3.13	0.077
	Urban	554 (51.1)	149 (55.8)	405 (49.6)		
	Rural	530 (48.9)	118 (44.2)	412 (50.4)		
Gender					0.01	0.932
	Male	514 (47.4)	126 (47.2)	388 (47.5)		
	Female	570 (52.6)	141 (52.8)	429 (52.5)		
Age, year					**43.57**	**<0.001**
	55~65	372 (34.3)	63 (23.6)	309 (37.8)		
	65~75	479 (44.2)	110 (41.2)	369 (45.2)		
	75~	233 (21.5)	94 (35.2)	139 (17.0)		
Educational level					**4.6**	**0.032**
	Junior school or below	927 (86.5)	236 (90.4)	691 (85.2)		
	Senior high school or above	145 (13.5)	25 (9.6)	120 (14.8)		
Occupation					0.14	0.710
	Employed or re-employed after retirement or seeking employment	166 (15.3)	39 (14.6)	127 (15.5)		
	Retired or unemployed	918 (84.7)	228 (85.4)	690 (84.5)		
Marriage					1.11	0.293
	Married	919 (84.8)	221 (82.8)	698 (85.4)		
	Unmarried/divorced/widowed	165 (15.2)	46 (17.2)	119 (14.6)		
BMI (kg/m^2^)					0.46	0.498
	<24.0	614 (56.6)	156 (58.4)	458 (56.1)		
	≥24	470 (43.4)	111 (41.6)	359 (43.9)		
Central obesity					1.04	0.308
	No	582 (55.0)	150 (57.7)	432 (54.1)		
	Yes	477 (45.0)	110 (42.3)	367 (45.9)		
Frequency of social activities					**24.04**	**<0.001**
	Once per year or less	275 (25.4)	98 (36.7)	177 (21.7)		
	More than once per year	809 (74.6)	169 (63.3)	640 (78.3)		
Depression					**20.09**	**<0.001**
	No	1041 (96.0)	244 (91.4)	797 (97.6)		
	Yes	43 (4.0)	23 (8.6)	20 (2.5)		
Sleep disturbances					**6.10**	**0.014**
	No	585 (54.1)	126 (47.6)	459 (56.3)		
	Yes	496 (45.9)	139 (52.5)	357 (43.8)		
Smoking					2.58	0.108
	No	882 (81.5)	208 (78.2)	674 (82.6)		
	Yes	200 (18.5)	58 (21.8)	142 (17.4)		
Hypertension					**15.58**	**<0.001**
	No	499 (46.0)	95 (35.6)	404 (49.5)		
	Yes	585 (54.0)	172 (64.4)	413 (50.6)		
Diabetes					2.71	0.100
	No	988 (91.6)	238 (89.1)	750 (92.4)		
	Yes	91 (8.4)	29 (10.9)	62 (7.6)		
Energy intake, kcal *		1517.5 ± 1282.4	1528.8 ± 1495	1513.8 ± 1205.8	−0.15	0.882

* Values are mean ± S and were statistically tested using a *t*-test. The numbers with missing values are 12 for educational level, 12 for occupation, 32 for BMI, 25 for central obesity, 3 for sleep disturbances, 2 for smoking, and 5 for diabetes, respectively. The statistical values and *p*-values in bold indicated that the differences were statistically significant.

**Table 2:** Odds ratio of MCI by both alcohol consumption and CADI.

Variables		CADI (Q1)	CADI (Q2)	CADI (Q3)	CADI (Q4)
Alcohol consumption	No	1.00	0.57 (0.37~0.90)	0.44 (0.27~0.73)	0.32 (0.16~0.63)
	Yes	1.43 (0.62~3.32)	1.60 (0.77~3.31)	1.53 (0.74~3.15)	0.35 (0.15~0.81)

## Data Availability

The data described in the manuscript, code book, and analytic code will not be made available due to privacy and ethical concerns with this survey. Detailed information on the dietary assessment method and MCI assessment methods can be provided to interested researchers in accordance with institutional and ethical guidelines. For specific inquiries, please contact the corresponding author: rhzhang@cdc.zj.cn.

## Supplementary Materials

The following supporting information can be downloaded at: https://www.mdpi.com/article/10.3390/nu17132111/s1, Table S1: Spearman correlation coefficients of daily nutrient intakes between FFQ and 24HRs; Table S2: Converted FFQ Food Composition Tables; Table S3: Frequency table of alcohol consumption and CDAI; Table S4: Sensitivity analysis of association between CDAI and MCI based on alcohol consumption. Table S5: Sensitivity analysis of association between CDAI and MCI based on alcohol consumption.

## Abbreviations

The following abbreviations are used in this manuscript:

AD	Alzheimer’s disease
BMI	Body Mass Index
CDAI	Composite dietary antioxidant index
CI	Confidence interval
DRIs	Dietary Reference Intakes
FCTs	Converted FFQ Food Composition Tables
FFQ	Food Frequency Questionnaire
GDS	Geriatric Depression Scale
MCI	Mild cognitive impairment
MOCA	Montreal Cognitive Assessment
MMSE	Mini-Mental State Examination
OR	Odds Ratio
PSQI	Pittsburgh Sleep Quality Index
RNI	Recommended Nutrient Intake
WFCT	Weighted Food Composition Table method
WHO	World Health Organization
24HRs	24 h dietary recall
