# Supplementary material for: Relationship of Composite Dietary Antioxidant Index vs. Alcohol Consumption with Mild Cognitive Impairment in the Elderly
Source: Nutrients. 2025 Jun 25;17(13):2111. doi: 10.3390/nu17132111 (PMC12251392; doi:10.3390/nu17132111)
Supplement: Supplementary file 1 [file nutrients-17-02111-s001.zip › nutrients-3648934-supplementary.pdf]

Supplementary Table S1 Spearman correlation coefficients of daily nutrient intakes between FFQ and 24HRs

**Nutrients**	**Spearman correlation coefficient**	***P***
Carbohydrate	0.16	<0.0001
Calcium	0.16	<0.0001
Carotene	0.07	0.035
Copper	0.17	<0.0001
Energy, kcal	0.28	<0.0001
Fat	0.32	<0.0001
Iron	0.19	<0.0001
Potassium	0.24	<0.0001
Magnesium	0.21	<0.0001
Manganese	0.10	0.002
Sodium	0.26	<0.0001
Niacin	0.32	<0.0001
Phosphorus	0.27	<0.0001
Protein	0.31	<0.0001
Riboflavin	0.29	<0.0001
Selenium	0.31	<0.0001
Thiamin	0.33	<0.0001
Total Vitamin A	0.13	<0.0001
Total Vitamin E	0.18	<0.0001
Vitamin C	0.18	<0.0001
Water	0.35	<0.0001
Zinc	0.29	<0.0001

Supplementary Table S2 Converted FFQ Food Composition Tables

Food group	Food name	Weight
1	Steamed rice (average value)	0.89819
1	Rice cake	0.10181
2	Noodles (standard wheat flour, cut noodles)	0.095389
2	Dumplings (pork and Chinese cabbage filling)	0.104704
2	Steamed buns (pork filling)	0.326705
2	Wheat flour (enriched flour, special first-class flour)	0.191281
2	Bread (average value)	0.129955
2	Steamed bread (average value)	0.151967
4	Pancake	0.219543
4	Fried dough sticks	0.780457
5	Instant noodles	1
6	Sweet potato (red heart) [yam, sweet potato]	0.633296
6	Bean vermicelli	0.366704
7	Taro [dasheen, eddo]	0.312694
7	Potato [potato]	0.687306
8	Millet	0.409659
8	Fresh corn	0.345992
8	Millet congee	0.244349
9	Germinated beans	0.23555
9	Soybean [soybean]	0.231021
9	Fresh green soybeans [green beans, vegetable soybeans]	0.264339
9	Dried green soybeans [green soybeans]	0.26909
10	Sweet soy milk	0.282251
10	Soy milk	0.717749
11	Tofu pudding [tofu powder]	1
12	Flavored dried tofu	0.131897
12	Fried bean curd puff	0.133118
12	Liaoning - style dried beancurd sheets	0.155219
12	Vegetable - filled dried tofu	0.171639
12	Firm tofu pudding [old - style tofu pudding]	0.071392
12	Vegetarian chicken	0.073772
12	Thin dried beancurd sheets [hundred - sheet tofu]	0.262962
13	String beans [kidney beans]	1
14	Snow peas	0.120913
14	Beans	0.317719
14	Fresh sword beans	0.130346
14	Dried cowpeas	0.128202
14	Dried lentils	0.302819
15	Canned whole tomatoes	1
16	Dried red pointed chili peppers	0.128741
16	Green pointed chili peppers	0.871259

17	Carrot	0.576593
17	Red carrot [golden carrot, caraway carrot]	0.423407
18	Zucchini	0.087168
18	Fresh cucumber [cucumber]	0.406629
18	Fresh pumpkin [pumpkin, cushaw]	0.506203
19	Pakchoi [bok choy]	1
20	Chinese cabbage (white stalk) [wong nga baak]	0.123455
20	White - stem celery [garden celery, medicinal celery]	0.246345
20	Celery stalk [garden celery, medicinal celery]	0.288081
20	Cabbage [round cabbage, head cabbage]	0.342119
21	Cantonese broccoli [broccoli]	0.178126
21	Cauliflower [cauliflower]	0.821874
22	Fresh asparagus lettuce [lettuce]	0.114973
22	Fresh water bamboo shoot [water bamboo shoot, water chestnut shoot]	0.232189
22	Fresh white radish [radish]	0.243676
22	Round white radish	0.409162
23	Garlic scapes	0.075854
23	Fresh onion [onion]	0.245672
23	Blanched leek [leek shoot]	0.429276
23	Garlic sprouts	0.249199
24	Fresh mushrooms	0.234306
24	Fresh kelp [sea cabbage]	0.205545
24	Dried laver	0.050142
24	Dried red mushrooms in general	0.097617
24	Dried fungus [black fungus, cloud ear]	0.10706
24	Fresh enoki mushrooms [intelligent mushrooms]	0.125007
24	Fresh shiitake mushrooms [mushroom, winter mushroom]	0.180323
25	Dehydrated cauliflower [dehydrated cauliflower]	0.045959
25	Dehydrated coriander	0.048378
25	Fried pumpkin seeds [white melon seeds]	0.118277
25	Fried peanuts	0.225614
25	Fresh peanuts [peanut, groundnut]	0.251652
25	Dried white bamboo shoot	0.310122
26	Pickled radish strips	0.099352
26	Dried radish	0.151696
26	Pickled mustard tuber	0.331103
26	Pickled turnip	0.417849
27	Fermented bean curd in wine sauce [wine - sauce bean curd]	0.205667
27	Broad - bean paste	0.794333
28	Pomelo [pummelo]	0.14979
28	Average value of citrus fruits	0.85021
29	Hami melon	0.256045

29	Muskmelon [cantaloupe]	0.743955
30	Winter jujube	0.038105
30	Longan	0.058868
30	Dragon fruit [pitaya, dragon fruit]	0.194338
30	Yangmei [Chinese bayberry, mountain bayberry]	0.708689
31	Raisins	0.136527
31	Dried litchi	0.182556
31	Dried jujube	0.680917
33	Fresh goat's milk	0.16129
33	Bright brand cow's milk	0.258065
33	Mengniu brand cow's milk	0.580645
35	Full - fat instant milk powder	0.250731
35	Full - fat milk powder	0.749269
37	Yogurt	1
38	Cheese [cheese]	1
39	Chocolate sundae ice cream	1
40	Domestic free - range chicken	0.119589
40	Black - boned chicken	0.307542
40	Chicken feet	0.57287
41	Lean pork	0.190195
41	Spare ribs (pork)	0.399205
41	Pork chop (pork)	0.4106
42	Pig's trotter	1
43	Beef (upper - brain, back meat)	0.096432
43	Frozen mutton	0.12054
43	Beef tenderloin [beef fillet]	0.12054
43	Beef tenderloin	0.265188
43	Dog meat	0.3973
44	Beijing roast duck	0.079248
44	Raw cured meat (with 68g fat)	0.084113
44	Sausage	0.090792
44	Cooked pig's knuckle	0.097469
44	Ham sausage	0.123531
44	Sauce - flavored spareribs	0.261706
44	Sauce - flavored duck	0.263141
45	Pig's small intestine	0.041095
45	Blood of female shelduck	0.05251
45	Pig's heart	0.065021
45	Pig's stomach	0.100783
45	Pig's blood	0.148855
45	Pig's liver	0.177134
45	Pig's large intestine	0.197712
45	Blood of white duck	0.21689
46	Crucian carp	0.301529

46	Silver carp [white amur, fatty fish, lotus - seed fish]	0.045603
46	Perch [perch flower]	0.060121
46	Naked fish	0.070447
46	Small yellow croaker	0.077973
46	Grass carp [white amur, grass - bag fish]	0.092362
46	Carp [carp]	0.111493
46	Cut hairtail	0.113548
46	Hairtail [belt fish, cutlass fish]	0.126925
47	Snail	0.039508
47	Prawn	0.041544
47	River shrimp [marsh shrimp]	0.043703
47	Red shrimp meat	0.052264
47	Dried shrimps [dried shrimp meat]	0.073498
47	River crab	0.079552
47	Prawn	0.11244
47	Sea shrimp	0.137492
47	Sea crab	0.207525
47	River shrimp	0.212473
48	White - shelled egg	0.071966
48	Fried poached egg	0.105511
48	Boiled salted duck egg	0.106656
48	Red - shelled egg (sampled in Beijing)	0.130642
48	Boiled egg	0.585224
49	Preserved egg (made from egg)	1
50	Soda crackers	0.285714
50	Sandwich soda biscuits	0.714286
51	Black - sesame glutinous rice balls	0.03947
51	Twisted dough sticks	0.050325
51	Barbecue - flavored potato chips	0.067757
51	Yellow cake	0.117753
51	Hollow crispy jujube	0.209017
51	Average - value cake	0.242084
51	Average - value biscuits	0.273594
52	Dried walnuts [walnut]	1
53	Chocolate	1
54	Kentucky Fried Chicken French fries	0.080645
54	Kentucky Fried Chicken chicken burger	0.258065
54	Kentucky Fried Chicken chicken wrap	0.290323
54	Kentucky Fried Chicken popcorn chicken	0.370968
55	Gujinggong Liquor (strong - flavor type, 38%)	0.377778
55	Hankou Baijiu (49.6 degrees)	0.622222
56	Huadiao wine (16.5%)	0.21832
56	Yellow rice wine	0.78168
57	Average - value beer	1

59	Coca - Cola	1
60	Suntory brand orange juice drink	0.461538
60	Coconut juice drink	0.538462
61	Black tea	0.470588
61	Green tea	0.529412
62	Coffee powder	1
64	fig	1

Supplementary Table S3 Food Frequency Questionnaire.

Food group		Frequency of Consumption (Single - choice)				Average Consumption per Time (grams or milliliters)
		Times per Day	Times per Week	Times per Month	Times per Year	
Staple Foods						
1	Rice (Steamed Rice/Rice Noodles)					grams
2	Non - Fried Pasta (Bread/Steamed Buns/Noodles/Dumplings), Excluding Instant Noodles					grams
3	Whole Grains (Whole - Wheat Bread, Buckwheat)					grams
4	Fried Pasta (Fried Dough Sticks, Fried Cakes, Twisted Dough Sticks)					grams
5	Instant Noodles					grams
6	Sweet Potatoes					grams
7	Potatoes/Taro/Sweet Potatoes					grams
8	Other Coarse Grains (Corn, Millet, Sorghum, Job's Tears, Rye)					grams
Beans						
9	Dried Soybeans (Soybeans, Green Beans or Black Beans)					grams
10	Soy Milk					milliliters
11	Soybean Powder					grams
12	Tofu, Dried Tofu Sheets					grams
13	Other Beans (Mung Beans/Adzuki Beans/String Beans)					grams
Vegetables						
14	Fresh Bean Vegetables (Peas, Snow Peas, Lentils, Cowpeas)					grams
15	Tomatoes					grams
16	Peppers (Red Peppers, Green Peppers, etc.)					grams
17	Carrots					grams
18	Gourd Vegetables (Cucumbers/Pumpkins/Zucchinis/Courgettes)					grams
19	Leafy Green Vegetables (Spinach/Rape/Water Spinach/Pakchoi)					grams
20	Chinese Cabbage and Other Leafy Vegetables (Cabbage/Celery/Lettuce, etc.)					grams
21	Cruciferous Vegetables (Broccoli, Cauliflower)					grams
22	Other Fresh or Frozen Vegetables (Bitter Gourds/Asparagus Lettuce/Radishes/Water Chestnuts/Bamboo Shoots), Excluding Canned, Dried, Fermented or Pickled Vegetables					grams
23	Onion and Garlic Vegetables (Garlic Sprouts/Blanched Leeks/Onions)					grams
24	Mushroom and Algae Vegetables (Mushrooms/Seaweeds)					grams
25	Dried Vegetables (Dehydrated Vegetables, Daylilies, Not Salted/Pickled/Fermented)					grams
26	Pickled Vegetables (Pickled Cabbage, Pickled Mustard Tubers, Pickled Cowpeas, etc.)					grams
27	Fermented Foods (Fermented Tofu, Bean Paste, Fermented Soybeans, Excluding Fermented Dairy Products, Beer, Soy Sauce and Vinegar)					grams
Fruits						

28	Orange - Colored Fruits (Oranges/Apricots/Persimmons/Mangoes/Papayas/Lemons)	grams
29	Watermelons/Muskmelons/Other Gourd - Shaped Fruits	grams
30	All Other Fresh/Frozen Fruits (Apples, Strawberries, Bananas, Kiwifruits, etc.)	grams
31	All Other Dried Fruits Except Preserved Fruits (Apples, Strawberries, Bananas, Red Dates, etc.)	grams
32	Preserved Fruits, Including Canned or Candied Fruits	grams
64	fig	grams
Dairy Products		
33	Whole - Fat Liquid Milk	milliliters
34	Low - Fat Liquid Milk/Skimmed Liquid Milk	milliliters
35	Whole - Fat Milk Powder	grams
36	Low - Fat Milk Powder/Skimmed Milk Powder	grams
37	Yogurt	grams
38	Cheese	grams
39	Ice Cream	grams
Meats		
40	Chicken, Duck, Goose, Pigeon, Quail (Fried/Non - Fried)	grams
41	Lean Pork	grams
42	Fatty Pork	grams
43	Beef/Lamb/Mutton/Other Unprocessed Meats	grams
44	Processed Meat Products (Sausages/Ham Sausages/Luncheon Meats)	grams
45	Poultry and Livestock Offal	grams
46	Fish (All Marine and Freshwater Fish)	grams
47	Shrimp, Crab or Other Seafood	grams
Eggs		
48	Fresh Eggs (Chicken Eggs/Duck Eggs/Quail Eggs)	grams
49	Preserved Eggs	grams
Snacks		
50	Salted Soda Crackers, Salted Mooncakes	grams
51	Sweet Cookies, Small Breads, Cakes, Pastries and Mooncakes	grams
52	Nuts (Peanuts/Seeds/Pumpkin Seeds/Watermelon Seeds/Other Seeds)	grams
53	Chocolate	grams
54	Fried Potato Chips/French Fries/Other Fried Snacks	grams
Alcoholic Beverages and Drinks		
55	Baijiu (All Types)	grams
56	Wine/Rice Wine/Millet Wine	grams
57	Beer	grams
58	Plain Water or Soda Water with No Additives	milliliters
59	Artificial Sweetened Beverages, Including Carbonated Drinks and Commercial Teas	milliliters
60	Sweetened Beverages, Such as Soft Drinks, Fruit Juice Drinks, Dairy - based Drinks, Sweet Water and Commercial Teas, Sports or Energy Drinks	milliliters
61	Tea	milliliters
62	Coffee (With or Without Sugar and/or Milk)	milliliters

63	100% Fruit Juice	milliliters

Supplementary Table S4 Frequency table of alcohol consumption and CDAI

	Alcohol consumption		
**Variables**	**No**	**Yes**	**Total**
**CADI(Q1)**	271(31.0)	38(18.1)	309(28.5)
**CADI(Q2)**	228(26.1)	52(24.8)	280(25.8)
**CADI(Q3)**	209(23.9)	50(23.8)	259(23.9)
**CADI(Q4)**	166(19.0)	70(33.3)	236(21.8)
**Total**	874(80.6)	210(19.4)	1084(100.0)

Supplementary Table S5 Sensitivity analysis of association between CDAI and MCI based on alcohol consumption

Variables		CADI(Q1)	CADI(Q2)	CADI(Q3)	CADI(Q4)
**Before adjustment**	**Alcohol consumption No**	1.00	0.57(0.36~0.89)	0.43(0.26~0.72)	0.30(0.15~0.63)
	**Alcohol consumption Yes**	1.00	1.48(0.52~4.23)	1.40(0.49~4.02)	0.34(0.09~1.21)
**After adjustment**	**Alcohol consumption No**	1.00	0.55(0.34~0.88)	0.46(0.28~0.76)	0.27(0.14~0.52)
	**Alcohol consumption Yes**	1.00	1.70(0.64~4.51)	0.84(0.31~2.23)	0.20(0.05~0.85)
